# Neurochemical Systems of the Retina Involved in the Control of Movement

**DOI:** 10.3389/fneur.2017.00324

**Published:** 2017-07-05

**Authors:** Gregory L. Willis, Christopher B. Freelance

**Affiliations:** ^1^Coliban Medical Centre, The Bronowski Institute of Behavioural Neuroscience, Kyneton, VIC, Australia

**Keywords:** dopamine, serotonin, melatonin, retina, Parkinson’s disease, intravitreal, motor function, microinjection

## Abstract

Recent studies have revealed that the retina may exert control over deep brain function and may be importantly involved in the etiology, progression, and treatment of disorders such as Parkinson’s disease (PD). While such a concept is uncharted territory and even less is known about the mechanism by which this might be achieved, this study was undertaken to determine how retinal dopamine (DA), serotonin (5-HT), and melatonin (MEL) neurotransmitter systems might be involved in the control of movement in their own right. To explore these further, intravitreal (IVIT) injections of DA, 5-HT, and MEL were made 0.5 or 3 h prior to testing horizontal and vertical movement in the open field as well as assessment on three motor tests used routinely to evaluate movement as a preclinical model of PD. The doses of DA (2 µl of 25 and 75 µg/µl), 5-HT (2 µl of 5 and 15 µg/µl), and MEL (2 µl of 5 µg/µl) were chosen because of previous work demonstrating an anatomically precise effect of these transmitters after they were injected directly into the brain. The postinjection times of testing were also chosen on the basis of previous intracerebral and IVIT work intimating the importance of the circadian cycle in determining the efficacy of such effects. 0.5 h after IVIT injection of DA at the 25 and 75 µg/µl doses, significant inhibition of motor function was observed. While IVIT injection of 10 or 30 µg of 5-HT also inhibited motor performance, this was significantly less than that seen with DA. In fact, IVIT injection increases motor performance compared to vehicle injection on some parameters. The IVIT injection of 10 µg of MEL facilitated motor function on many parameters compared to DA, 5-HT, and vehicle injection. When rats were tested 3 h after IVIT injection, the inhibition of vertical movement was also observed compared to controls. The present results illustrate that specific retinal neurotransmitter systems participate in the normal control of bodily motor function. The possible involvement of these systems in movement disorders such as PD is the subject of ongoing research.

## Introduction

Recent evidence has emerged proposing that the retina is functionally linked to nigro-striatal dopamine (NSD) system control over motor function. This has been illustrated in a number of ways, including an intimately detailed proposition that there is a neurological system running between the retina and the pineal gland that communicates with crucial hypothalamic and midbrain structures during its course of passage (1). In addition, the simple pharmacological illustration that the intravitreal (IVIT) injection of microgram quantities of anti-Parkinsonian drugs can restore impaired NSD system function, in a pattern emulating the course of circadian function, is an intriguing finding (2). In fact, the observed behavioral recovery occurs even though the variables of anatomical distance and compartmentalization of the retina render the two events anatomically unrelated. From a traditional perspective whereby impaired motor function of Parkinson’s disease (PD) is reflexively attributed to impaired NSD system function, such a finding would be simply regarded as inexplicable. However, a deeper examination of both clinical and experimental manifestations of this disease reveal that impaired visual function is shared and that dopamine (DA) deficiency of the retina is, in fact, a bi-product of the disease as well (3–5). What remains a further mystery is why DA deficiency of the retina occurs in parallel with the development of PD and how these two events may be anatomically, physiologically, and etiologically linked. Indeed, such findings require a deeper examination as to the definition of PD, its etiology, and the role of the retina in movement. While the role of the NSD system in PD sits as the core proposition upon which the entire arsenal for treating this disease has been developed (6), this presents problems with interpretation since DA deficiency in the retina and NSD system occur concomitantly (3–5). At the very least, to imply cause and effect, good scientific procedure would dictate that retinal and NSD involvement would each have to be studied individually to determine their participation in the possible sequelae of events underlying this disorder.

Recent experimental and clinical reports have demonstrated retinal involvement in the etiology (7) and symptomatic improvement of PD (2). While such work has examined neurotransmitter function in the pathological state of DA deficiency, work on the role of retinal neurotransmitters in motor performance of non-PD rats with normal retinal function has not been examined. Therefore, it was hypothesized that if IVIT administration of neurotoxic (7) and therapeutic agents (2) can induce and repair experimental PD by their effect upon the retina, then the IVIT administration of neurotransmitters should also induce change in gross motor function in normal animals. Given that DA, serotonin (5-HT), and melatonin (MEL) occur naturally in mammalian retina (8–15), these neuroactive substances were selected for testing in this study and were injected in doses so minute that if any alteration in motor performance was observed then such changes could not be attributed to diffusion of the drug into CNS sites known to be involved in motor control (16–18).

## Materials and Methods

42 male, Sprague-Dawley rats were obtained from the Bronowski Institute colony and were housed individually in plastic boxes with wire mesh tops. Standard food pellets (Clarke King^®^/Barastock^®^) were made available *ad lib* from a feeding grid on the top of each cage while tap water was also made available through the feeding grid. Animals ranged in weight from 250 to 350 g at the commencement of the experiment. Room temperature was maintained at 22°C ± 2° with a 12 h light: 12 h dark cycle with lights on at 0700 h. The room was illuminated with two fluorescent tubes with the intensity of light within each cage averaging 250 lx during the light phase of the light/dark cycle. No light was detected in the housing facility during the dark phase. This study was carried out in accordance with the Australian Code for the Care and Use of Animals for Scientific Purposes and was approved and monitored by the Animal Experimentation Ethics Committee of the Bronowski Institute of Behavioral Neuroscience.

### Study Design

#### Study I: IVIT DA

After habituation into the colony for at least 7 days, all animals were handled by all experimenters prior to commencing the formal part of each study to minimize stress. Prior to determining the effects of the test drugs or vehicle solutions on motor performance, all rats underwent control measurement at least 6 days prior to drug testing and these served as the baseline measurements for all DA experiments. In the *first experiment*, two groups of rats consisting of seven animals per group were employed with the first group receiving a bilateral IVIT DA (50 µg in 2 µl) injection, while the second group received a 2 µl IVIT injection of vehicle, bilaterally. These rats were tested 30 min after injection just prior to assessment of motor function during the light phase of the light/dark cycle (see Behavioral Measures). For dark phase testing, all animals were injected again with either DA (50 µg in 2 µl) or vehicle (2 µl) and then tested on all motor tests allowing at least 48 h between injections. Rats were then given 6 days to recover from the first study and then the same paradigm was repeated for the *second experiment* using the same animals, except that 3 h was permitted between the time of injection and the time of assessment of motor function. The animals were again used in a *third experiment* following the same paradigm as described for the first study with the exception that a dose of 150 µg in 2 µl (bilaterally) was employed for the IVIT injection. As with previous studies, 48 h elapsed between light phase and dark phase testing while the amount of rest time between the second and third experiments was 6 days.

#### Study II: IVIT 5-HT

In a *fourth experiment*, 14 more rats were tested for baseline performance and then divided into two groups of rats with seven animals per group. One group was injected bilaterally with IVIT 5-HT (10 µg in 2 µl), while the second group of rats were injected with 2 µl of vehicle. As with Study I, motor function was again examined 30 min after IVIT injection during the light phase and dark phase testing, allowing 2 days of recovery before performing the dark phase test. The same rats were used in a *fifth experiment* that was procedurally identical with experiment four. All parameters were the same with the exception that 5-HT was used for IVIT injection in a dose of 30 µg in 2 µl (bilaterally) during the light and dark phases of the L/D cycle.

#### Study III: IVIT MEL

In the *sixth experiment*, a new group of seven animals were given a bilateral IVIT MEL (10 µg in 2 µl) injection, while a second group of seven received the control bilateral 2 µl IVIT vehicle injection. The paradigm applied was identical to experiment five with all rats tested 0.5 h after IVIT injection during both the light phase and the dark phase of the L/D cycle.

### Drugs and Solutions for IVIT Injections

Dopamine hydrochloride was acquired from Sigma-Aldrich (St. Louis, MO, USA; product no. H8502) and was prepared for IVIT injection at concentrations of 50 and 150 µg/2 μl by dissolving it in 0.09% sterile saline solution (Pfizer). 5-HT creatinine sulfate complex (Sigma Chemicals; product no. H7752) and mixed in the concentrations of 10 and 30 µg/2 µl and dissolved in 0.09% isotonic saline. MEL (Sigma-Aldrich; product no. M5250) was prepared at the concentrations of 10 and 30 µg/2 μl in 75% solution of dimethyl sulfoxide (DMSO). Vehicle injections for all other drugs were made with the 0.09% saline solution with the exception of MEL where 2 µl of a 75% DMSO solution was employed. New solutions of drug were prepared immediately prior to each injection and were kept on ice and protected from light and were discarded immediately at the end of each injection session. All concentrations of drug employed in both studies were chosen on the basis of previous work describing a localized effect of these compounds when injected by the intracerebral route (19–23). In consideration of the dose, volume of injectate and potency of effect from previous studies, any findings in this study would preclude the possibility that they represent the effect of a leakage of test substances into sensitive brain areas after IVIT administration.

### IVIT Injections

Injections into the vitreous humor were made with the aid of a 10 µl syringe fitted with a 26 g needle 75 mm in length. The needle was fitted with a colored plastic sleeve exposing 3 mm of the tip to allow the experimenter to gage the depth of needle insertion into the center of the vitreal mass. Rats were first placed in a clear Perspex induction chamber (200 mm × 300 mm × 400 mm) fitted with a base constructed from heavy gage plastic netting above a 25 mm layer of cotton baton that served to absorb and hold the anesthetic. Isoflurane inhalation anesthetic (Attane-Bomac: 1 mg/ml) was employed by placing approximately 10–20 ml into the absorbent cotton surface just prior to placing the animal into the induction chamber. Exposure of the animal for 60–100 s induced a state of deep anesthesia that lasted 60–90 s, thereby permitting the injection of the test substances and vehicle bilaterally into the vitreous. To keep the preparation clean, the surrounding fur was swabbed with ethanol prior to injection. To facilitate injection into the lateral aspect of the eye, light pressure was placed on the caudal surface of the eye using a sterile, gloved, tip of the index finger to cause it to become a maneuverable exophthalmic mass and thereby permit the experimenter to gently apply counter pressure when the needle was inserted into the vitreous mass. In each case, the appropriate drug was injected bilaterally in a volume of 2 µl, commencing with the left eye. Postinjection, the area was gently swabbed with sterile isotonic saline and a drop of antibiotic ointment [Amacin^®^ eye and ear ointment (Jurox Pty Ltd., Rutherford, NSW, Australia)] was placed on the cornea as a prophylactic. Rats were held and kept warm until they were able to ambulate on their own before being returned to their individual cage.

### Behavioral Measures

Independent variables were measured in the same animals during the light phase and the dark phase of the L/D cycle commencing between 1000–1500 hours and again at 2000–0100 hours, respectively. For each drug dose tested, the effects of IVIT drug administration on motor performance were tested in the sequence of light phase testing followed by two rest days. If more than one dose of a drug was tested, then rats were rested for at least 6 days before subsequent testing commenced. This paradigm has been implemented previously (2, 17, 20, 22) as it reliably depicts the relationship in movement during the light phase versus the dark phase of the L/D cycle and is indicative of normal circadian patterns in this species.

Locomotion (horizontal movement) and rearing (vertical movement) were measured with the aid of a 900 mm (length) × 500 mm (width) × 300 mm (height) PVC box fitted with machine vision with motion detection capabilities as implemented previously (2, 7, 24, 25). The number of movements within the horizontal plane and the number of rearing-associated movements in the vertical plane during each 10-min test session were measured and recorded with the aid of specialized software. A series of three motor reflex tests were performed immediately at the conclusion of the open field test (24). These tests included the latency to retract the left and right front limbs when they were elevated 25 mm from the table surface, the latency to step up or down from a raised platform when the rear torso was elevated 25 mm and the latency to ambulate outside of a 90 mm × 170 mm rectangle. These tests are derivations of those described originally by Balagura et al. (26) and have been used extensively to characterize the features of experimental PD (24). The test chamber and all surfaces and each apparatus were thoroughly washed between the testing of each animal to avoid scent contamination that may cause distraction during testing. Testing during the dark phase of the light/dark cycle was performed under low intensity red light with all sources of illumination masked by implementing red barrier filters (Lee Filters no. 106 “Primary Red”).

### Statistical Analysis

Due to the high degree of between-subject variability that commonly exists in horizontal and vertical movements, different scores were calculated by subtracting performance after IVIT injection with baseline performance during control measurements taken prior to commencing the formal study. The latency to retract, step, and ambulate in drug- and vehicle-treated groups were analyzed as raw scores. A one-way analysis of variance (ANOVA) with *post hoc* LSD was first employed to determine whether there was a main effect across all drug-treated animals at 30 min post IVIT injection for each parameter examined for the light phase and the dark phase of the light/dark cycle. The Levene test for homogeneity of variance was performed and, if significant, the Games–Howell (GH) *post hoc* test was implemented assuming inequality of variance. A one-way ANOVA was also used to examine differences between each drug and vehicle-treated animals tested 3 h after the IVIT injection. If Levene’s test returned as statistically significant, then non-parametric independent samples Mann–Whitney *U* (MWU) test was employed for comparing each drug-injected group to their vehicle-injected counterparts. Given that the hypothesis permitted prediction of the direction of the expected outcome, a one-tailed test was employed with exact significance. The confidence levels were chosen *a priori* and set at 5% to depict a significant effect, while *p*-values ranging from 0.051 to 0.099 depicted a significant trend. All vehicle-/saline-injected controls for each parameter for all animals tested 30 min after IVIT injection were combined after analysis revealing that they did not differ prior to commencing IVIT drug testing. Analysis of control performance after IVIT administration of 75% DMSO was not significantly different to that of rats receiving IVIT control injection of isotonic saline. This permitted the combining of all control sessions into one group for statistical comparison across all drug-treated groups tested at 30 min post IVIT injection. All statistical analyses were performed using SPSS Statistics v.24 (IBM, Armonk, NY, USA).

## Results

**Figure 1:**
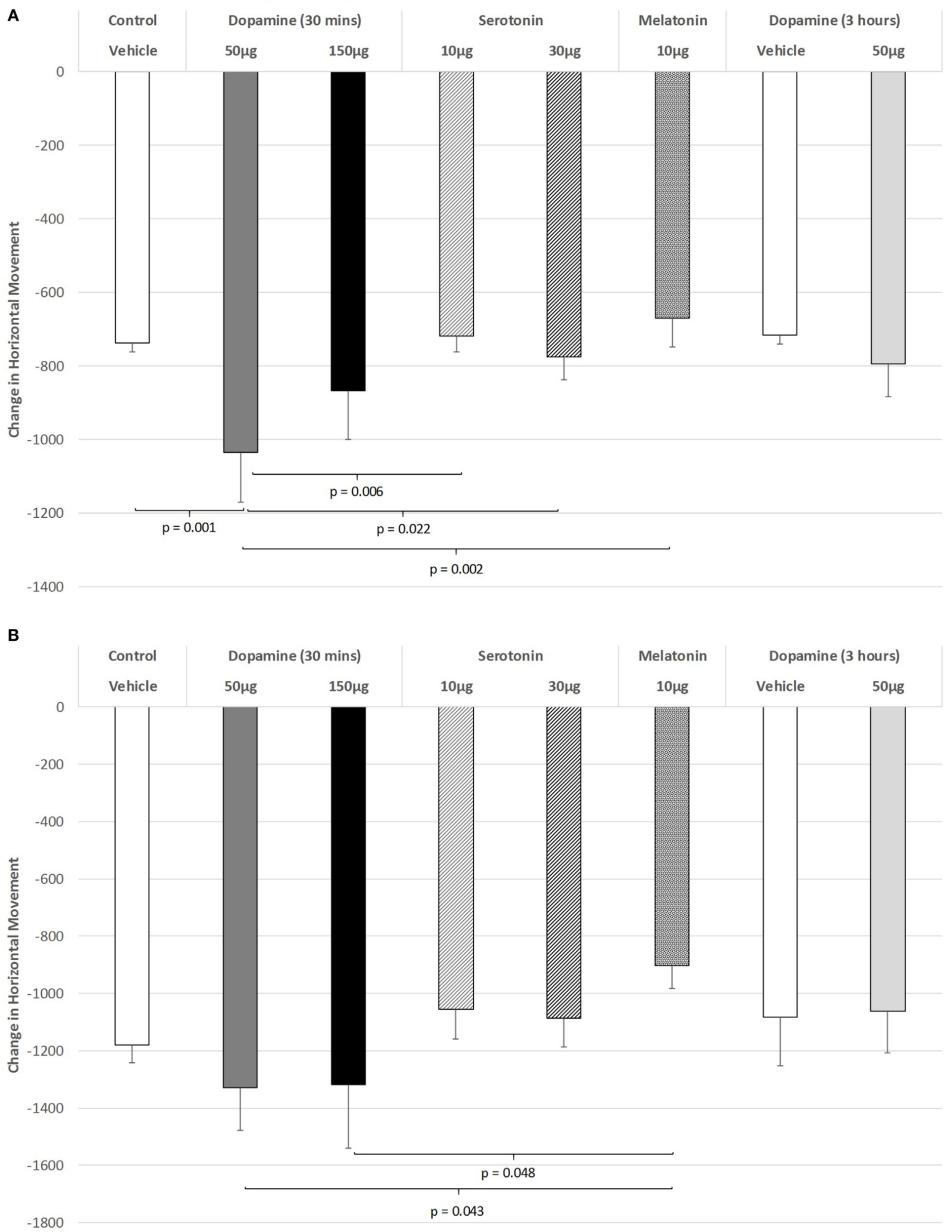
The effect of intravitreal (IVIT) dopamine (DA), serotonin, melatonin, and saline (vehicle) on horizontal movement during the **(A)** light phase and **(B)** dark phase of the light/dark cycle. Difference scores were obtained by subtracting the drug injection performance from the non-injected control performance. Rats were tested 30 min after IVIT injection in all groups with the exception of an additional group receiving 2 µl of control solution bilaterally and a second group receiving 50 µg of DA 3 h before testing. Values in all control animals tested 0.5 h after injection were combined into a single control group as they did not differ significantly from each other. The T-bars represent the SEM.

As shown in Figure 1A, there was a significant impairment of horizontal movement during the light phase of the L/D cycle in rats receiving IVIT injection of 50 µg of DA compared to rats receiving IVIT injections of isotonic saline [ANOVA (main effect): df = 5.69; *F* = 3.117, *p* = 0.014; *post hoc* LSD multiple comparisons: *p* = 0.001]. The 50 µg DA injection group was also more impaired than the 10 and 30 µg 5-HT groups (*p* = 0.006 and *p* = 0.022, respectively) as well as the 10 µg of MEL (*p* = 0.002). A weak, significant trend of improvement was also seen after 10 µg of MEL compared to 150 µg of DA (*p* = 0.081).

Figure 1B illustrates the changes in horizontal movement during the dark phase of the L/D cycle after IVIT injections of DA. While a significant main effect was not observed (ANOVA: df = 5.69; *F* = 1.345, *p* = 0.257), *post hoc* LSD analysis revealed that rats injected with the 50 or 150 µg doses of DA differed significantly from those injected with 10 µg of MEL, as they were less impaired (*p* = 0.043 and *p* = 0.048, respectively). There was also a strong trend for improvement after 10 µg of MEL compared to the control group (*p* = 0.067).

**Figure 2:**
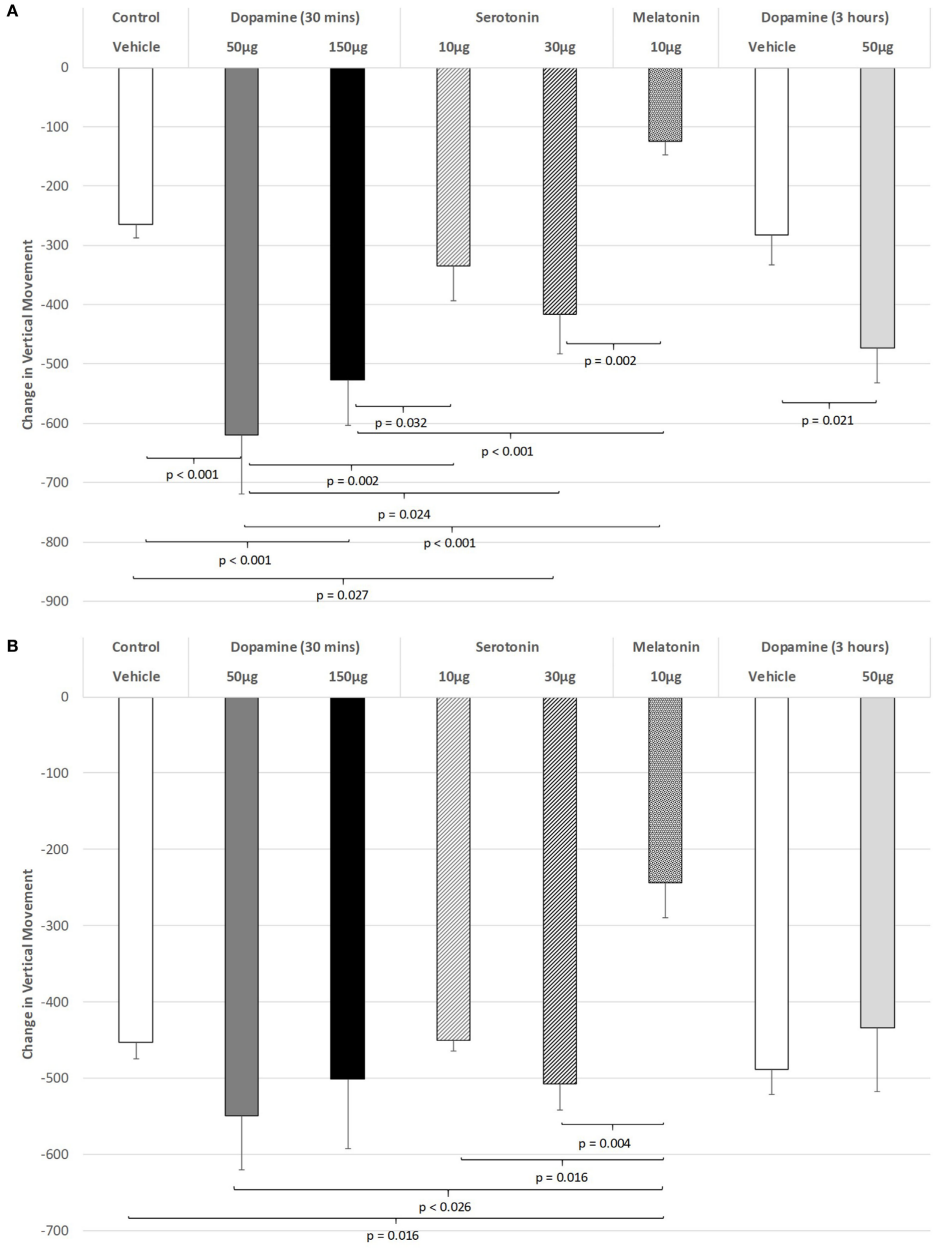
The effect of intravitreal (IVIT) dopamine (DA), serotonin, melatonin, and saline (vehicle) on vertical movement during the **(A)** light phase and **(B)** dark phase of the light/dark cycle. Difference scores were obtained by subtracting the drug injection performance from the non-injected control performance. Rats were tested 30 min after IVIT injection in all groups with the exception of an additional group receiving 2 µl of control solution bilaterally and a second group receiving 50 µg of DA 3 h before testing. Values in all control animals tested 0.5 h after injection were combined into a single control group as they did not differ significantly from each other. The T-bars represent the SEM.

The effect of IVIT injections on vertical movement during the light phase are expressed in Figure 2A. ANOVA revealed a significant main effect (df = 5.69; *F* = 10.062, *p* < 0.001), with LSD *post hoc* comparisons illustrating significant differences between 50 µg DA compared to control injection (*p* < 0.001), 10 µg of 5-HT (*p* = 0.002), 30 µg of 5-HT (*p* = 0.024), and 10 µg of MEL (*p* < 0.001). The 150 µg DA group tested at 30 min were also found to be more severely impaired on this parameter compared to controls (*p* < 0.001), 10 µg of 5-HT (*p* = 0.032), and the 10 µg of MEL groups (*p* < 0.001). Rats injected with 30 µg of 5-HT showed significantly less vertical movement during the light phase when compared to controls (*p* = 0.027) and those injected with 10 μg of MEL (*p* = 0.002). Rats injected with 30 µg of DA and tested at 3 h were significantly impaired at that time compared to their saline-injected counterparts (*p* = 0.021). There was also a strong trend for MEL-injected rats to display improved motor performance compared to saline-injected controls (*p* = 0.052).

Intravitreal injections of various compounds during the dark phase of the L/D cycle (Figure 2B) were without effect on vertical movement, with the exception of the MEL-injected group. With the main effect showing a high level of significance (ANOVA: df = 5.69, *F* = 3.628, *p* = 0.006), robust decreases were observed between MEL and the following groups: control (*p* = 0.016), 50 µg DA (*p* < 0.026), 10 µg of 5-HT (*p* = 0.016), and 30 µg of 5-HT (*p* = 0.004) (all *p* values were derived from *post hoc* GH analysis assuming unequal variance: Levene Statistic, *p* = 0.012).

**Figure 3:**
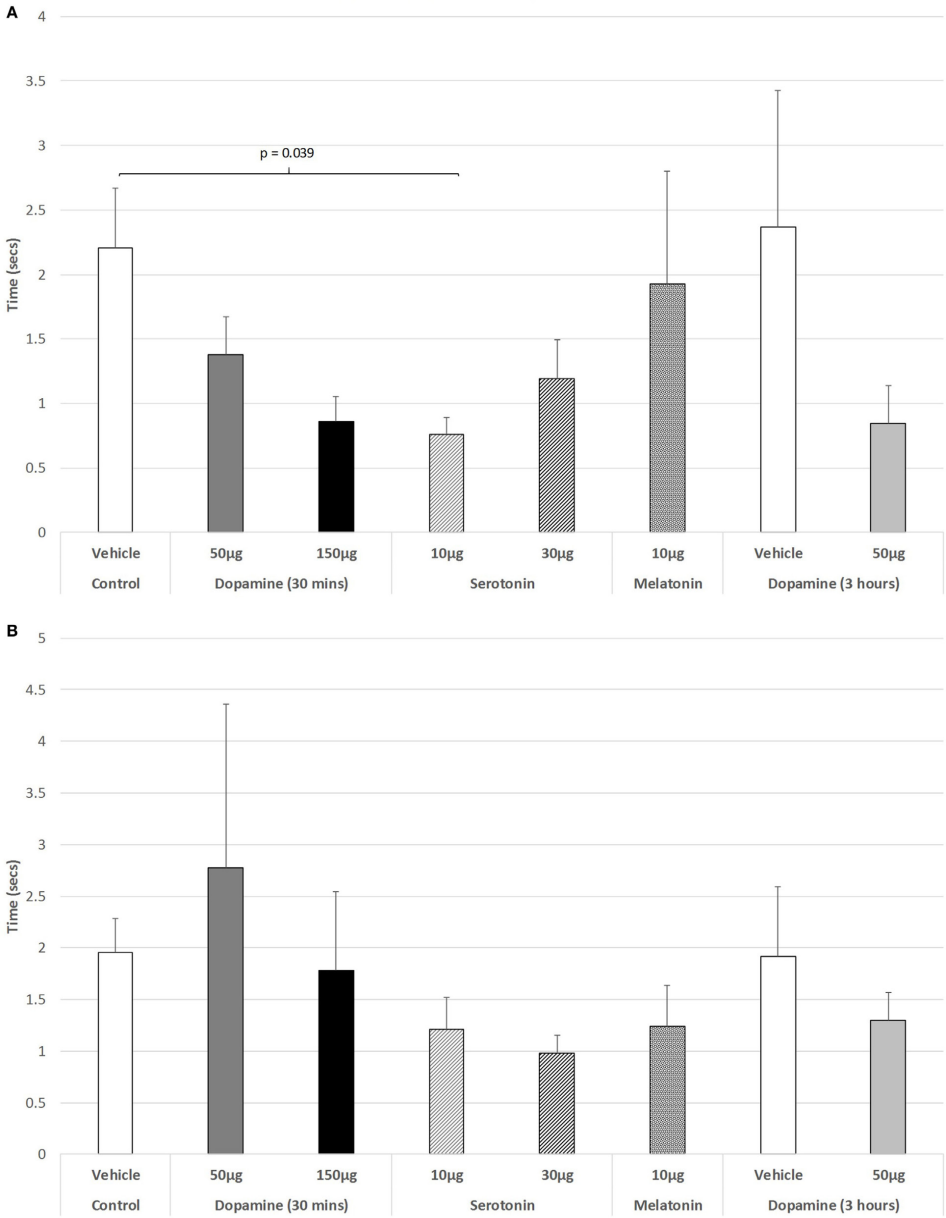
The effect of intravitreal (IVIT) dopamine (DA), serotonin, melatonin, and saline (vehicle) on latency to retract a limb during the **(A)** light phase and **(B)** dark phase of the light/dark cycle. Rats were tested 30 min after IVIT injection in all groups with the exception of an additional group receiving 2 µl of control solution bilaterally and a second group receiving 50 µg of DA 3 h before testing. Values in all control animals tested 0.5 h after injection were combined into a single control group as they did not differ significantly from each other. The T-bars represent the SEM.

The effect of IVIT injections on latency to retract a limb were virtually without effect during both the light and dark phases of the L/D cycle. ANOVA revealed non-significant effects (Figure 3A: light phase: df = 3.139; *F* = 1.023, *p* = 0.407; Figure 3B: dark phase: df = 5.139, *F* = 0.801, *p* = 0.551). The only significant difference between groups in the ability to retract a limb during the light phase was after the IVIT injection of 10 µg of 5-HT compared to controls (*p* = 0.039, *post hoc* GH analysis assuming unequal variance: Levene Statistic, *p* = 0.028).

**Figure 4:**
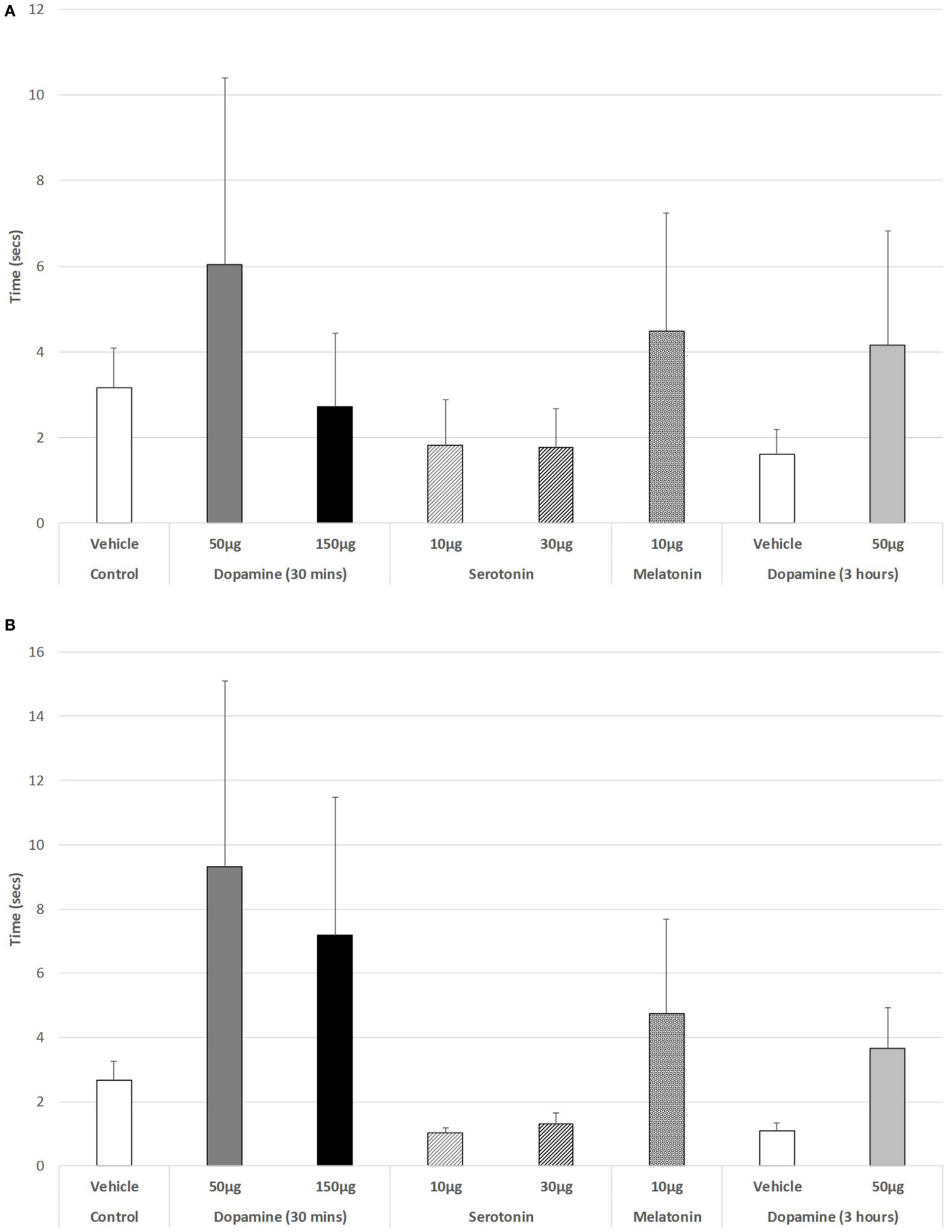
The effect of intravitreal (IVIT) dopamine (DA), serotonin, melatonin, and saline (vehicle) on latency to step up or down during the **(A)** light phase and **(B)** dark phase of the light/dark cycle. Rats were tested 30 min after IVIT injection in all groups with the exception of an additional group receiving 2 µl of control solution bilaterally and a second group receiving 50 µg of DA 3 h before testing. Values in all control animals tested 0.5 h after injection were combined into a single control group as they did not differ significantly from each other. The T-bars represent the SEM.

As shown in Figure 4A, there was no significant change in the ability to step up or down from a raised platform during the light phase of the L/D cycle in any of the drug-treated groups [ANOVA (main effect): df = 3.41, *F* = 0.447, *p* = 0.721], nor as revealed with the *post hoc* LSD analysis. However, during the dark phase (Figure 4B), a weak significant trend was revealed (ANOVA: df = 5.69, *F* = 2.193, *p* = 0.066). *Post hoc* GH analysis revealed a weak trend of improvement in the ability to step down for 10 µg 5-HT versus controls (*p* = 0.098), with a Levene Statistic of *p* < 0.001.

**Figure 5:**
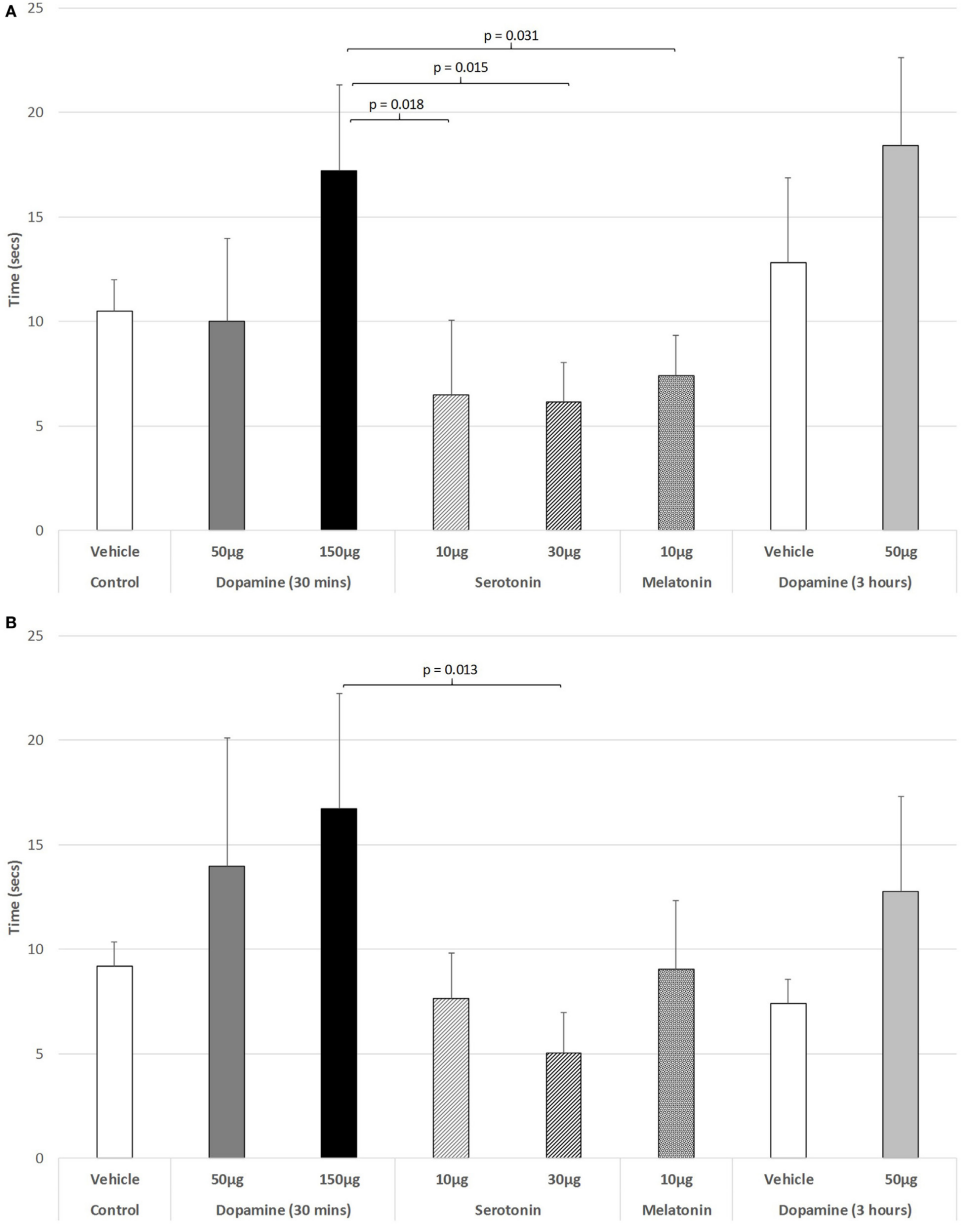
The effect of intravitreal (IVIT) dopamine (DA), serotonin, melatonin, and saline (vehicle) on latency to ambulate from a prescribed area during the **(A)** light phase and **(B)** dark phase of the light/dark cycle. Rats were tested 30 min after IVIT injection in all groups with the exception of an additional group receiving 2 µl of control solution bilaterally and a second group receiving 50 µg of DA 3 h before testing. Values in all control animals tested 0.5 h after injection were combined into a single control group as they did not differ significantly from each other. The T-bars represent the SEM.

Figure 5A illustrates that there was a trend to increase the latency to ambulate after IVIT injection during the light phase (ANOVA: df = 5.69, *F* = 1.778, *p* = 0.130). On *post hoc* analysis, the group receiving 150 µg DA was significantly slower than those receiving 10 µg of 5-HT (*p* = 0.018), 30 µg of 5-HT (*p* = 0.015), or 10 µg of MEL (*p* = 0.031). A strong trend was also revealed, with 150 µg DA being slower than controls (*p* = 0.055). During the dark phase (Figure 5B), no significant main effect was detected (ANOVA: df = 5.69, *F* = 1.812, *p* = 0.123), while there was no difference between any of the drug-treated groups as revealed by *post hoc* GH analysis (Levene Statistic *p* < 0.001).

For the rats measured 3 h after receiving an IVIT injection during the light phase, only limb retraction was significantly faster in rats injected with 50 µg DA (ANOVA: df = 1.13, *F* = 6.998, *p* = 0.021) compared to controls. Furthermore, a significant, weak trend toward slowing the ability to step down during the dark phase was also seen in these rats treated with 150 µg DA 3 h prior to testing (Levene’s test for homogeneity *p* = 0.005, MWU test *p* = 0.097).

## Discussion

The present results demonstrate that the injection of minute quantities of DA, 5-HT, and MEL into the vitreous can alter horizontal and vertical movement and motor reflex control. These substances were chosen for study on the basis of their functional importance in the retina as well as their role in normal motor function and in pathological conditions such as PD (8–15). Furthermore, this study is an important advance on previous work whereby IVIT administration of minute quantities of the DA precursor l-DOPA and the MEL receptor antagonist ML-23 significantly repaired experimental PD (2). In our attempt to examine the role of the retina as the initiatory locus for light to stimulate the circadian system (27, 28), the present findings illustrate the importance of retinal systems in exerting control over deep brain modulation of motor function. That the dose of drug employed in this study is rendering its effect directly upon the retina is intimated by two lines of evidence. First, the dose of drugs are so low that when similar, or even larger, doses are injected directly into brain sites suspected to have an effect on various physiological and behavioral parameters, no effect is seen when such injections are translocated only a few millimeters away (18). This is an important concept given that the distance from brain sites actively involved in motor control in the rat are not only located 25 mm away from the retina, but they are also contained within a separate body compartment. Second, recent studies illustrated that herbicides, insecticides, and neurotoxins that were injected into the vitreous of the rat can induce a state closely resembling experimental PD (7). The doses of such toxins, when injected directly into the brains of this species, have no effect when placed 2 or 3 mm from the intended anatomical target (i.e., the NSD system). These published results also dovetail with the present findings to suggest which neurochemical systems in the retina may be involved. From this, we draw the tentative conclusion that the retina is the location where anti-Parkinsonian medication might be rendering at least some of its therapeutic effect or even inducing adverse side effects.

Given the exploratory nature of this study, the underlying hypothesis would be bidirectional in predicting outcome. This is based on previous work concerning the role of circadian, NSD, and retina DA systems in movement demonstrating that the ability of DA to provide therapeutic relief or to enhance motor impairment in PD is dependent upon: the dose and type of DA replacement administered, the anatomical site involved, and the motor parameter examined. For example, inhibition of motor performance has been reported in experimental PD after systemic l-DOPA and other forms of DA replacement (29). This effect is seen in experimental models of PD, in intact rats and in rats with compromised circadian function (1, 30). Clinically, l-DOPA has been shown to exacerbate motor impairment, cause dysphonia, impair swallowing, aggravate depression, and enhance tremor in PD patients (31–40). Of the substances and doses tested, DA in both doses caused more inhibition of motor performance during the light phase than the dark phase of the L/D cycle and was never effective in reversing the deficits in motor performance at either of the two doses employed but caused them only to worsen. While 5-HT did not cause such inhibition of motor performance, the effect of 5-HT in either dose was not as inhibitory as DA. Motor performance after MEL was better during the dark phase than the light phase of the L/D cycle, with this dual effect between DA and MEL being similar to that seen in previous studies (2). This suggests participation of retina as part of circadian system in the control of deep brain modulation of motor function. The present and previous results (1) reveal a complex system regulating motor performance in the normal and pathological brain, intimating that the effect of therapeutic drugs used for treating PD will now have to be screened for their effects upon the retina as well as their ability to replace deficient DA in the NSD system. Further research will help to reveal the participation of each in the neurological matrix governing motor control in vertebrates.

One of the principal reasons for undertaking this study was to determine the role of the retina, as the initiatory site of the response to light, in the circadian system in motor function. To achieve this, injections were made prior to testing during the light or dark phase of the L/D cycle and the difference in response between the light phase and the dark phase was remarkable. For example, the inhibitory effect of DA on motor function when compared to 5-HT or MEL was much more potent during the light phase than during the dark phase. MEL, on the other hand, was more effective at improving horizontal and vertical movements during the light phase and the dark phase, but was particularly effective during the light phase for vertical movement. This is consistent with the circadian-related therapeutic effects that were observed when MEL antagonism was achieved *via* the IVIT administration of MEL receptor antagonist ML-23 during the dark phase, while this effect was less potent when applied during the light phase (41, 42). Similarly, when l-DOPA was administered *via* the IVIT route, it was more effective when administered during the light phase compared to the dark phase (2). The previous IVIT administration of neurotoxins showed similar light/dark differentiation (7), providing further support for the hypothesis that the circadian system is involved in the etiology of PD (7, 43). Traditional clinical work with PD supports the contention of circadian involvement, in that symptom expression varied with changes in the L/D cycle, as well as the increasing interest in the use of light treatment in this disorder (27, 28, 44, 45).

While differences in light phase versus dark phase were consistent with other studies and are understandable in relation to results from previous studies, the effect of IVIT injections on some motor parameters (i.e., horizontal and vertical movements, and ambulation) while others parameters (i.e., latency to retract and to step) were not affected reveals a differential sensitivity of the systems involved in different aspects of motor control. In previous work (7), we have found that latency to retract a limb is a particularly sensitive motor measure compared to other measures, such as stepping or latency to ambulate. Why this showed no significant changes across the different conditions in this study is yet to be explained. However, the present studies are pioneering in that the doses employed are extremely low in comparison to those needed to evoke a response after systemic or even intracerebral doses commonly employed. Future studies examining dose dependency may reveal which systems are involved and their relative sensitivity to the applied doses, that may help to differentiate which systems control what aspects of motor function in more detail.

It is interesting to note that the neurotransmitters and hormone studied in this study do not appear to render an effect on motor function as potent as those achieved previously with the precursor l-DOPA (2) or the MEL receptor antagonist ML-23 (41, 42). While this might represent a potency problem that may be resolved with further dose–response studies, it might alternatively suggest that these could represent more effective means acting on other mechanisms for treating the disease, combined with a more effective route of administration. This is consistent with the explanation as to why l-DOPA remains the most widely used therapeutic for PD (6). In addition, PD patients are characterized as having comorbid problems with sight (3, 4, 46, 47) further suggesting that the visual system is, in fact, mediating the observed effects of l-DOPA therapy. It is interesting to note further that when PD patients are administered anti-PD drugs, it is often reported that their vision also improves with such treatment (48). When considering the relationship between the retina and deep brain, it is worth bearing in mind that the retina is the only sensory organ that embryologically emerges from brain, whereas all other sensory systems are derived from cells that extend connections into the brain. While pharmaceutical companies spend extensively on studies devoted to “targeting” anatomical sites in the CNS that are candidates for treatment intervention, this study confirms previous findings suggesting that in PD, the retina provides direct access to deep brain function and in particular motor control. Results from this study support the suggestion that the retina is the site from which PD may commence and progress (2) and ultimately it is a locus from which it might be treated. By this line of reasoning, a more causal relationship between retinal function and PD is evident and from this emerges a novel hypothesis that needs to be more fully explored. The implementation of IVIT injections to target retinal systems with minute drug doses may serve to dramatically redefine the effective therapeutic dose and reduce side effects of DA replacement. With this, the need for more complex and invasive procedures that detract from the quality of life in PD patients would be diminished.

## Ethics Statement

This study was carried out in accordance with the Australian Code for the Care and Use of Animals for Scientific Purposes. The protocol was approved and the experiment monitored by the Animal Experimentation Ethics Committee of The Bronowski Institute of Behavioural Neuroscience.

## Author Contributions

GW was responsible for design and conception of the work. GW and CF were responsible for acquisition, analysis, and interpretation of the data and for preparing the manuscript.

## Conflict of Interest Statement

The authors declare that the research was conducted in the absence of any commercial or financial relationships that could be construed as a potential conflict of interest.
